# The Impact of Pathway Database Choice on Statistical Enrichment Analysis and Predictive Modeling

**DOI:** 10.3389/fgene.2019.01203

**Published:** 2019-11-22

**Authors:** Sarah Mubeen, Charles Tapley Hoyt, André Gemünd, Martin Hofmann-Apitius, Holger Fröhlich, Daniel Domingo-Fernández

**Affiliations:** ^1^Department of Bioinformatics, Fraunhofer Institute for Algorithms and Scientific Computing (SCAI), Sankt Augustin, Germany; ^2^Bonn-Aachen International Center for IT, Rheinische Friedrich-Wilhelms-Universität Bonn, Bonn, Germany

**Keywords:** pathway enrichment, benchmarking, databases, machine learning, statistical hypothesis testing

## Abstract

Pathway-centric approaches are widely used to interpret and contextualize -*omics* data. However, databases contain different representations of the same biological pathway, which may lead to different results of statistical enrichment analysis and predictive models in the context of precision medicine. We have performed an in-depth benchmarking of the impact of pathway database choice on statistical enrichment analysis and predictive modeling. We analyzed five cancer datasets using three major pathway databases and developed an approach to merge several databases into a single integrative one: MPath. Our results show that equivalent pathways from different databases yield disparate results in statistical enrichment analysis. Moreover, we observed a significant dataset-dependent impact on the performance of machine learning models on different prediction tasks. In some cases, MPath significantly improved prediction performance and also reduced the variance of prediction performances. Furthermore, MPath yielded more consistent and biologically plausible results in statistical enrichment analyses. In summary, this benchmarking study demonstrates that pathway database choice can influence the results of statistical enrichment analysis and predictive modeling. Therefore, we recommend the use of multiple pathway databases or integrative ones.

## Introduction

As fundamental interactions within complex biological systems have been discovered in experimental biology labs, they have often been assembled into computable pathway representations. Because they have proven immensely useful in the analysis and interpretation of -*omics* data when coupled with algorithmic approaches (e.g., gene set enrichment analysis, GSEA), academic and commercial groups have generated and maintained a comprehensive set of databases during the last 15 years (Bader et al., 2006). Examples include KEGG, Reactome, WikiPathways, NCIPathways, and Pathway Commons (Schaefer et al., 2008; Cerami et al., 2011; Kanehisa et al., 2016; Slenter et al., 2017; Fabregat et al., 2018).

However, these databases tend to differ in the average number of pathways they contain, the average number of proteins per pathway, the types of biochemical interactions they incorporate, and the subcategories of pathways that they provide (e.g., signal transduction, genetic interaction, and metabolic) (Kirouac et al., 2012; Türei et al., 2016). Pathways are often also described at varying levels of detail, with diverse data types and with loosely defined boundaries (Domingo-Fernández et al., 2018). Nonetheless, most pathway analyses are still conducted exclusively by employing a single database, often chosen in part by researchers’ preferences or previous experiences (e.g., bias towards a database previously yielding good results and ease of use of a particular database) (**Table 1**). Notably, the selection of a suitable pathway database depends on the actual biological context that is investigated, yet KEGG remains severely overrepresented in published -omics studies. This raises concerns and motivates the consideration of multiple pathway databases or, preferably, an integration over several pathways resources.

**Table 1:** Number of publications citing major pathway resources for pathway enrichment in PubMed Central (PMC), 2019. To develop an estimate on the number of publications using several pathway databases for pathway enrichment, SCAIView (http://academia.scaiview.com/academia; indexed on 01/03/2019) was used to conduct the following query using the PMC corpus: “<pathway resource>” AND “pathway enrichment”.

Type	Pathway resource	Publications
**Primary**	**KEGG**	27,713
	**Reactome**	3,765
	**WikiPathways**	651
**Integrative**	**MSigDB**	2,892
	**ConsensusPathDB**	339
	**Pathway Commons**	1,640

Several integrative resources have been developed, including meta-databases [e.g., Pathway Commons (Cerami et al., 2011), MSigDB (Liberzon et al., 2015), and ConsensusPathDB (Kamburov et al., 2008)] that enable pathway exploration in their corresponding web applications and integrative software tools [e.g., graphite (Sales et al., 2018), PathMe (Domingo-Fernandez et al., 2019), and OmniPath (Türei et al., 2016)] designed to enable bioinformatics analyses. By consolidating pathway databases, these resources have attempted to summarize major reference points in the existing knowledge and demonstrate how data contained in one resource can be complemented by data contained in others. Thus, through their usage, the biomedical community has benefitted from comprehensive overviews of pathway landscapes which can then make for more robust resources highly suited for analytic usage.

The typical approach to combine pathway information with -omics data is *via* statistical enrichment analysis, also known as pathway enrichment. The task of navigating through the continuously developing variants of enrichment methods has been undertaken by several recent studies which benchmarked the performance of these techniques (Bayerlová et al., 2015; Ihnatova et al., 2018; Lim et al., 2018) and guide users on the choice for their analyses (Fabris et al., 2019; Reimand et al., 2019). While Bateman et al. (2014) examined the impact of choice of different subsets of MSigDB on GSEA, it remains unclear what broader impact an integrative pathway meta-database would have for statistical enrichment analysis. Additionally, the overlap of pathways within the same integrative database can induce biases (Liberzon et al., 2015), specifically when conducting multiple testing correction *via* the popular Benjamini–Hochberg method (Benjamini and Hochberg, 1995) that supposes independence of statistical tests. This issue is of particular concern for large-scale meta-databases such as MSigDB.

The aim of this work is to systematically investigate the influence of alternative representations of the same biological pathway (e.g., in KEGG, Reactome, and WikiPathways) on the results of statistical enrichment analysis *via* three common methods: the hypergeometric test, GSEA, and signaling pathway impact analysis (SPIA) (Fisher, 1992; Subramanian et al., 2005; Tarca et al., 2008) using five The Cancer Genome Atlas (TCGA) datasets (Weinstein et al., 2013). In addition, we also show that pathway activity-based patient classification and survival analysis *via* single-sample GSEA (ssGSEA; Barbie et al., 2009) can be impacted by the choice of pathway resource in some cases. As a solution, we propose to integrate different pathway resources *via* a method where semantically analogous pathways across databases (e.g., “Notch signaling pathway” in KEGG and “Signaling by NOTCH” pathway in Reactome) are combined. This approach exploits the pathway mappings and harmonized pathway representations described in our previous work (Domingo-Fernández et al., 2018; Domingo-Fernandez et al., 2019). We demonstrate that when aided by our integrative pathway database, it is possible to better capture expected disease biology than with individual resources, and to sometimes obtain better predictions of clinical endpoints. Our entire analytic pipeline is implemented in a reusable Python package (pathway_forte; see *Materials and Methods*) to facilitate reproducing the results with other databases or datasets in the future.

## Materials and Methods

In the first two subsections, we describe the pathway resources and the clinical and genomic datasets we used in benchmarking. The following sections then outline the statistical enrichment analysis and predictive modeling conducted in this study. Finally, in the last two subsections, we describe the statistical methods and the software implemented to conduct the benchmarking.

### Pathway Databases

#### Selection Criteria

Numerous viable pathway databases have been made available to infer biologically relevant pathway activity (Bader et al., 2006). In this work, we systematically compared three major ones (i.e., KEGG, Reactome, and WikiPathways) as the subset of databases to benchmark. The rationale for the inclusion of these databases was twofold: firstly, these databases are open-sourced, well-established, and highly cited in studies investigating pathways associated with variable gene expression patterns in different sets of conditions (**Table 1**). Secondly, we expected distinctions between these databases to be strong enough to observe variable results of enrichment analysis and patient classification, yet these databases also contain a reasonable number of equivalent pathways such that objective comparisons could be made, as outlined in our previous work (Domingo-Fernández et al., 2018).

#### Data Retrieval and Processing

In order to systematically compare results yielded by different databases, we retrieved the contents of KEGG, Reactome, and WikiPathways using ComPath (Domingo-Fernández et al., 2018) and converted it into the Gene Matrix Transposed (GMT) file format. Generated networks encoded in Biological Expression Language (BEL; Slater, 2014) were retrieved using PathMe (Domingo-Fernández et al., 2019).

**Figure 1:**
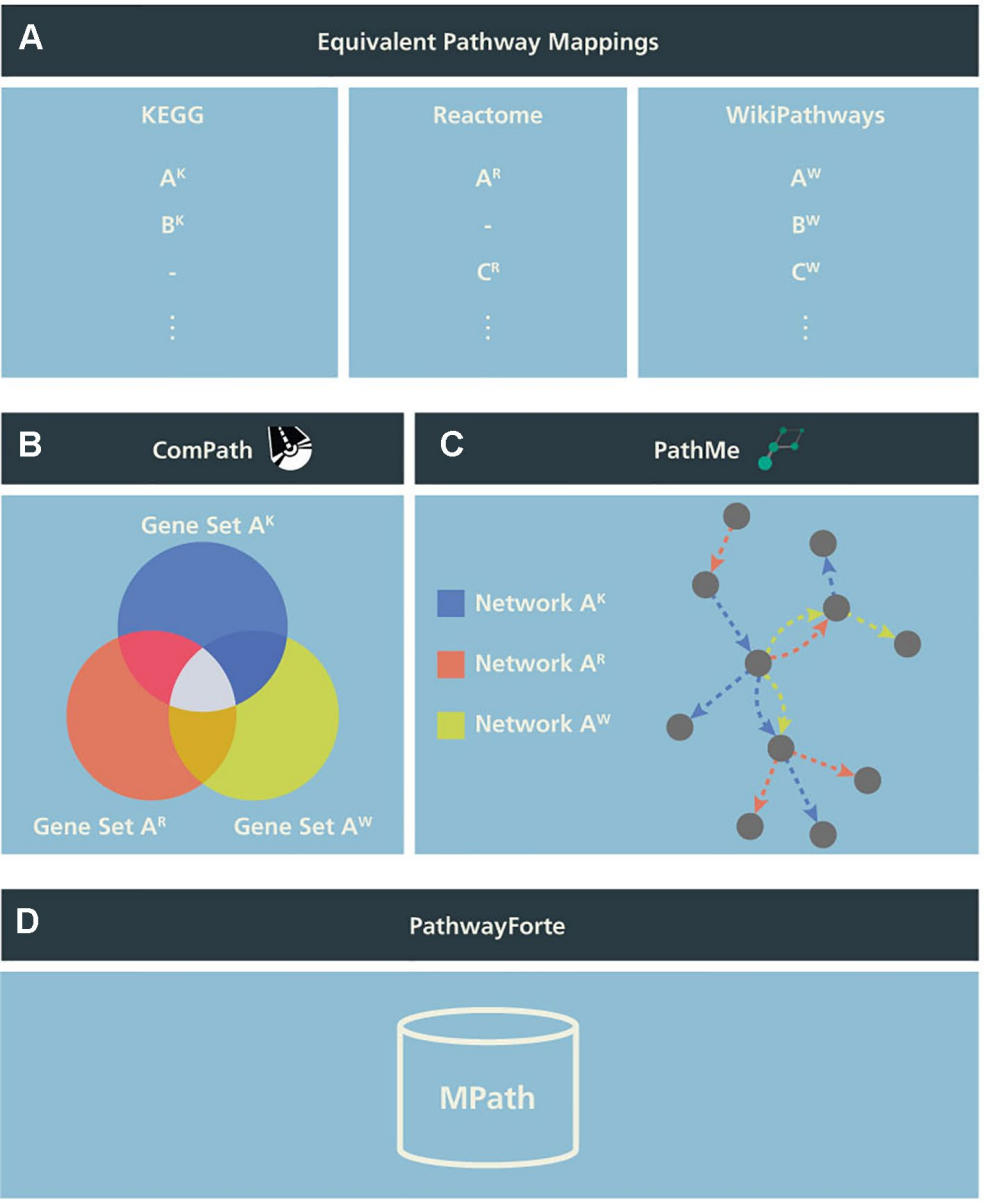
Schema illustrating the generation of MPath. The curated pathway mapping catalog is depicted in **(A)**, which links equivalent pathways from different resources. Pathways that are shared across two resources are referred to as pathway analogs (i.e., Pathway A in Reactome and Pathway A′ in KEGG) and pathways that are shared across all three resources are referred to as “super pathways” (i.e., Pathway A in KEGG, Pathway A′ in Reactome, and Pathway A″ in WikiPathways). **(B)** Using these mappings, gene sets of equivalent pathways from different resources can be combined, ensuring key molecular players from the different resources are included. **(C)** Similarly, network representations of the pathways can be overlaid to generate more comprehensive pathways. **(D)** Finally, both the combined gene sets and networks representations are included in MPath. Note that pathways that are exclusive to a single database are included in MPath unchanged.

To test the potential utility of an integrative pathway resource, we used equivalent pathways across the three databases that were manually curated in our previous work (Domingo-Fernández et al., 2018; see our earlier publication for further details). In the following, we call these “pathways analogs” or “equivalent pathways” (**Figure 1A**), while we call a pathway found as analogous across all KEGG, Reactome, as well as WikiPathways a “super pathway”.

In a second step, we merged equivalent pathways by taking the graph union with respect to contained genes and interactions (**Figures 1B, C**). We have also described this step in more detail in our earlier work (Domingo-Fernandez et al., 2019).

The set union of KEGG, Reactome, and WikiPathways, while taking into account pathway equivalence, gave rise to an integrative resource to which we refer as *MPath* (**Figure 1D**). By merging equivalent pathways, MPath contains a fewer number of pathways than the sum of all pathways from all primary resources. In total, MPath contains 2,896 pathways, of which 238 are derived from KEGG, 2,119 from Reactome, and 409 from WikiPathways, while another 129 pathways are pathway analogs and 26 are super pathways.

We next compared the latest versions of pathway gene sets from KEGG, Reactome, WikiPathways, and MPath with pathway gene sets from MSigDB, a highly cited integrative pathway database containing older versions of the KEGG and Reactome gene sets (Liberzon et al., 2015). We downloaded KEGG and Reactome gene sets from the curated gene set (C2) collection of MSigDB (http://software.broadinstitute.org/gsea/msigdb/collections.jsp #C2; version6.2; July 2018). Detailed statistics on the number of pathways from each resource are presented in **Table S1**.

### Clinical and Genomic Data

We used five widely used datasets acquired from TCGA (Weinstein et al., 2013), a cancer genomics project that has catalogued molecular and clinical information for normal and tumor samples (**Table 2**). TCGA data were retrieved through the Genomic Data Commons (GDC; https://gdc.cancer.gov) portal and cBioportal (https://www.cbioportal.org) on 14-03-2019. RNA-seq gene expression data subjected to an mRNA quantification analysis pipeline for BRCA, KIRC, LIHC, OV, and PRAD TCGA datasets were queried, downloaded, and prepared from the GDC through the R/Bioconductor package, TCGAbiolinks (R version: 3.5.2; TCGAbiolinks version: 2.10.3) (Colaprico et al., 2015). The data were preprocessed as follows: gene expression was quantified by the number of reads aligned to each gene and read counts were measured using HTSeq and normalized using fragments per kilobase of transcript per million mapped reads upper quartile (FPKM-UQ). HTSeq raw read counts also subject to the GDC pipeline were similarly queried, downloaded, and prepared with TCGAbiolinks. Read count data downloaded for the BRCA, KIRC, LIHC, and PRAD datasets were processed to remove identical entries, while unique measurements of identical genes were averaged. The differential gene expression analysis of cancer versus normal samples was performed using the R/Bioconductor package, DESeq2 (version 1.22.2). Genes with adjusted *p* value < 5% were considered significantly dysregulated. For all downloaded data, gene identifiers were mapped to HGNC gene symbols (Povey et al., 2001), where possible. To obtain additional information on the survival status and time to death, or censored survival times of patients, patient identifiers in the TCGA datasets were mapped to their equivalent identifiers in cBioPortal. Additionally, cancer subtype classifications or the PRAD and BRCA datasets were retrieved from the GDC. We would like to note that although there are other cohorts available (e.g., COAD and STAD) containing all of these modalities, we did not include them in this analysis because of the limited number of samples they contain (i.e., less than 300 patients). Detailed statistics of all five datasets are presented in **Table 2**.

**Table 2:** Statistics of the five TCGA cancer datasets used in this work.

Cancer type	TCGA abbreviation	Tumor samples	Normal samples	Surviving patients	Deceased patients
**Breast invasive carcinoma**	BRCA	1,102	113	946	153
**Kidney renal clear cell carcinoma**	KIRC	538	72	365	173
**Liver hepatocellular carcinoma**	LIHC	371	50	240	130
**Prostate adenocarcinoma**	PRAD	498	52	498	10
**Ovarian cancer**	OV	374	0	143	229

The statistics correspond to those retrieved from the GDC portal and cBioportal on 14-03-2019. Longitudinal statistics of survival data are presented in **Figure S1**.

### Pathway Enrichment Methods

**Figure 2:**
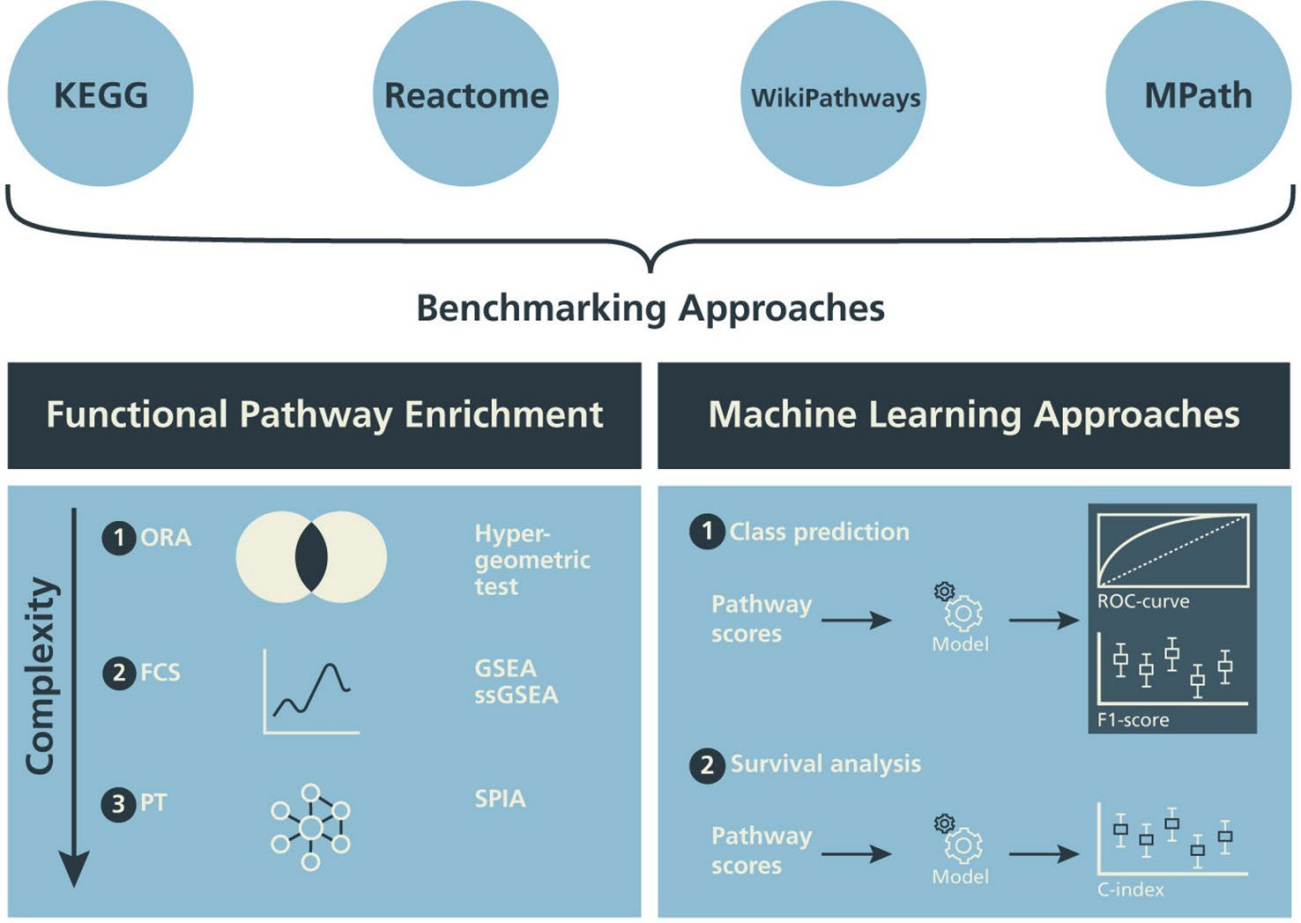
Design of the benchmarking schema. The influence of alternative pathway databases on the results of statistical pathway enrichment (left) and machine learning classification tasks (right) are compared.

In this subsection, we describe three different classes of pathway enrichment methods that we tested: 1) statistical overrepresentation analysis (ORA); 2) functional class scoring (FCS); and 3) pathway topology (PT)-based enrichment (**Figure 2**) (Khatri et al., 2012; García-Campos et al., 2015; Fabris et al., 2019).

#### Overrepresentation Analysis

We conducted pathway enrichment using genes that exhibited a *q* value <0.05 using a one-sided Fisher’s exact test (Fisher, 1992) for each of the pathways in all pathway databases. We consider a pathway to be significantly enriched if its *q* value is smaller than 0.05 after applying multiple hypothesis testing correction with the Benjamini–Yekutieli method under dependency (Benjamini and Yekutieli, 2001).

#### Functional Class Scoring Methods

We selected GSEA, one of the most commonly used FCS methods (Subramanian et al., 2005). We performed GSEA with the Python package, GSEApy (version 0.9.12; https://github.com/zqfang/gseapy), using normalized RNA-seq expression quantifications (FPKM-UQ) obtained for the BRCA, KIRC, LIHC, and PRAD datasets containing both normal and tumor samples (**Table 2**). All genes were ranked by their differential expression based on their log_2_ fold changes. Query gene sets for GSEA included pathways from KEGG, Reactome, WikiPathways, and MPath. GSEA results were filtered to include pathway gene sets with *p* values below 0.05 and a minimum gene set size of 10 or a maximum gene size of 3,000. Similarly, GSEApy was used to perform ssGSEA (Barbie et al., 2009) (**Table S2**) to acquire sample-wise pathway scores using FPKM-UQ for BRCA, KIRC, LIHC, OV, and PRAD datasets, irrespective of phenotype labels (Barbie et al., 2009). Datasets were filtered to only include normalized expression data for genes found in the pathway gene sets of KEGG, Reactome, WikiPathways, and MPath and then used for ssGSEA. Expression data were ranked and sample-wise normalized enrichment scores were obtained.

#### Pathway Topology-Based Enrichment

To evaluate PT-based methods, we selected the well-known and highly cited SPIA method (Tarca et al., 2008) for two main reasons: firstly, the guidelines outlined by a comparative study on topology-based methods (Ihnatova et al., 2018) recommend the use of SPIA for datasets with properties similar to TCGA (i.e., possessing two well-defined classes, full expression profiles, many samples, and numerous differentially expressed genes). Secondly, SPIA has been reported to have a high specificity while preserving dependency on topological information (Ihnatova et al., 2018). Because the R/Bioconductor’s SPIA package only contains KEGG pathways, we converted the pathway topologies from the three databases used in this work to a custom format in a similar fashion as graphite (Sales et al., 2018) (**Supplementary Text**). We declared significance for SPIA-based pathway enrichment, if the Bonferroni corrected *p* value was <5%.

#### Evaluation Based on Enrichment of Pathway Analogs

To better understand the impact of database choice, we compared the raw *p* value rankings (i.e., before multiple testing correction) of pathway analogs across each possible pair of databases (i.e., in KEGG and Reactome, Reactome and WikiPathways, and WikiPathways and KEGG) and in each statistical enrichment analysis (i.e., hypergeometric test, GSEA, and SPIA) with the Wilcoxon signed-rank test. It assessed the average rank difference of the pathway analogs and reported how significantly different the results were for each database pair. Importantly, we only tested statistical enrichment of the analogous pathways in order to avoid statistical biases due to differences in the size of pathway databases.

### Machine Learning

ssGSEA was conducted to summarize the gene expression profile mapping to a particular pathway of interest within a given patient sample, hence resulting in a pathway activity profile for each patient. We then evaluated the different pathway resources with respect to three machine learning tasks:

1. Prediction of tumor vs. normal
2. Prediction of known tumor subtype
3. Prediction of overall survival

#### Prediction of Tumor vs. Normal

The first task was to train and evaluate binary classifiers to predict normal versus tumor sample labels. This task was conducted for four of the five TCGA datasets (i.e., BRCA, KIRC, LIHC, and PRAD), while OV, which only contains tumor samples, was omitted. We performed this classification using a commonly used elastic net penalized logistic regression model (Zou and Trevor, 2005). Prediction performance was evaluated *via* a 10 times repeated 10-fold stratified cross-validation. Importantly, tuning of elastic net hyper-parameters (*l*_1_, *l*_2_ regularization parameters) was conducted within the cross-validation loop to avoid over-optimism (Molinaro et al., 2005).

#### Prediction of Tumor Subtype

The second task was to train and evaluate multi-label classifiers to predict tumor subtypes using sample-wise pathway activity scores generated from ssGSEA. This task was only conducted for the BRCA and PRAD datasets, similar to the work done by Lim et al. (2018), because the remaining three datasets included in this work lacked subtype information. From the five breast cancer subtypes present in the BRCA dataset by the PAM50 classification method (Sorlie et al., 2001), we included four subtypes (i.e., 194 Basal samples, 82 Her2 samples, 567 LumA samples, and 207 LumB samples). These four were selected as they constitute the agreed-upon intrinsic breast cancer subtypes according to the 2015 St. Gallen Consensus Conference (Coates et al., 2015) and are also recommended by the ESMO Clinical Practice Guidelines (Senkus et al., 2015). For the PRAD dataset, evaluated subtypes included 151 ERG samples, 27 ETV1 samples, 14 ETV4 samples, 38 SPOP samples, and 87 samples classified as other (Cancer Genome Atlas Research Network, 2014). Similar to the approach by Graudenzi et al. (2017), support vector machines (SVMs) (Cortes and Vapnik, 1995) were used for subtype classification by implementing a one-versus-one strategy in which a single classifier is fit for each pair of class labels. This strategy transforms a multi-class classification problem into a set of binary classification problems. We again used a 10 times repeated 10-fold cross-validation scheme, and the soft margin parameter of the linear SVM was tuned within the cross-validation loop *via* a grid search. We assessed the multi-class classifier performance in terms of accuracy, precision, and recall.

#### Prediction of Overall Survival

The third task was to train and evaluate machine learning models to predict overall survival of cancer patients. For this purpose, a Cox proportional hazards model with elastic net penalty was used (Tibshirani, 1997; Friedman et al., 2010). Prediction performance was evaluated on the basis of five TCGA datasets (i.e., BRCA, LIHC, KIRC, OV, and PRAD) (**Table 2**) using the same 10 times repeated 10-fold nested cross-validation procedure as described before. The performance of the model was assessed by Harrell’s concordance index (c-index; Harrell et al., 1982), which is an extension of the well-known area under receiver operating characteristic (ROC) curve for right censored time-to-event (here: death) data.

#### Statistical Assessment of Database Impact on Prediction Performance

To understand the degree to which the observed variability of area under the ROC curve (AUC) values, accuracies, and c-indices could be explained by the actually used pathway resource, we conducted a two-way analysis of variance (ANOVA). The ANOVA model had the following form:

performance∼database+dataset+database×dataset

We then tested the significance of the database factor *via* an *F* test. In addition, we performed Wilcoxon tests analysis to understand specific differences between databases in a dataset-dependent manner.

### Software Implementation

The workflow presented in this article consists of three major components: 1) the acquisition and preprocessing of gene set and pathway databases; 2) the acquisition and preprocessing of experimental datasets; and 3) the re-implementation or adaptation of existing analytical pipelines for benchmarking. We implemented these components in the pathway_forte Python package to facilitate the reproducibility of this work, the inclusion of additional gene set and pathway databases, and to include additional experimental datasets.

The acquisition of KEGG, MSigDB, Reactome, and WikiPathways was mediated by their corresponding Bio2BEL Python packages (Hoyt et al., 2019; https://github.com/bio2bel) in order to provide uniform access to the underlying databases and to enable the reproduction of this work as they are updated. Each Bio2BEL package uses Python’s *entry points* to integrate in the previously mentioned ComPath framework in order to support uniform preprocessing and enable the integration of further pathway databases in the future, without changing any underlying code in the pathway_forte package. The network preprocessing defers to PathMe (Domingo-Fernandez et al., 2019; https://github.com/pathwaymerger). Because it is based on PyBEL (Hoyt et al., 2018; https://github.com/pybel), it is extensible to the growing ecosystem of BEL-aware software.

While the acquisition and preprocessing of experimental datasets is currently limited to a subset of TCGA, it is extensible to further cancer-specific and other condition-specific datasets. We implemented independent preprocessing pipelines for several previously mentioned datasets using extensive manual curation, preparation, and processing with the pandas Python package (McKinney, 2010; https://github.com/pandas-dev/pandas). Unlike the pathway databases, which were amenable to standardization, the preprocessing of each new dataset must be bespoke.

The re-implementation and adaptation of existing analytical methods for functional enrichment and prediction involved wrapping several existing analytical packages (**Table S3**) in order to make their application programming interfaces more user-friendly and to make the business logic of the benchmarking more elegantly reflected in the source code of pathway_forte. Each is independent and can be used with any combination of pathway database and dataset. Finally, all figures presented in this paper and complementary analyses can be generated and reproduced with the Jupyter notebooks located at https://github.com/pathwayforte/results/.

Ultimately, we wrapped each of these components in a command line interface (CLI) such that the results presented in each section of this work can be generated with a corresponding command following the guidelines described by Grüning et al. (2019). The scripts for generating the figures in this manuscript are not included in the main pathway_forte, but rather in their own repository within Jupyter notebooks at https://github.com/PathwayForte/results.

The source code of the pathway_forte Python package is available at https://github.com/PathwayForte/pathway-forte, its latest documentation can be found at https://pathwayforte.readthedocs.io, and its distributions can be found on PyPI at https://pypi.org/project/pathway-forte.

The pathway_forte Python package has a tool chain consisting of pytest (https://github.com/pytest-dev/pytest) as a testing framework, coverage (https://github.com/nedbat/coveragepy) to assess testing coverage, sphinx (https://github.com/sphinx-doc/sphinx) to build documentation, flake8 (https://github.com/PyCQA/flake8) to enforce code and documentation quality, setuptools (https://github.com/pypa/setuptools) to build distributions, pyroma (https://github.com/regebro/pyroma) to enforce package metadata standards, and tox (https://github.com/tox-dev/tox) as a build tool to facilitate the usage of each of these tools in a reproducible way. It leverages community and open-source resources to improve its usability by using Travis-CI (https://travis-ci.com) as a continuous integration service, monitoring testing coverage with Codecov (https://codecov.io), and hosting its documentation on Read the Docs (https://readthedocs.org).

### Hardware

Computations for each of the tasks were performed on a symmetric multiprocessing (SMP) node with four Intel Xeon Platinum 8160 processors per node with 24 cores/48 threads each (96 cores/192 threads per node in total) and 2.1-GHz base/3.7-GHz Turbo Frequency with 1,536-GB/1.5-TB RAM (DDR4 ECC Reg). The network was 100 GBit/s Intel OmniPath, storage was 2× Intel P4600 1.6-TB U.2 PCIe NVMe for local intermediate data and BeeGFS parallel file system for Home directories. **Table 3** provides a qualitative description of the memory and time requirements for each task.

**Table 3:** A qualitative description of the computational costs of the analyses performed.

Task	Relative memory usage	Timescale
ORA	Low	Seconds
GSEA	Medium	Minutes
ssGSEA	Very high	Hours
Prediction of tumor vs. normal	Medium	Minutes
Prediction of known tumor subtype	Medium	Minutes
Prediction of overall survival	Medium	Hours

Performing ssGSEA required on the scale of 100 GB of RAM for some dataset/database combinations, while the other tasks could be run on a modern laptop with no issues.

## Results

The results of the benchmarking study have been divided into two subsections for each of the pathway methods described above. We first compared the effects of database selection on the results of functional pathway enrichment methods. In the following subsection, we benchmarked the performance of the pathway resources on the various machine learning classification tasks conducted.

### Benchmarking the Impact on Enrichment Methods

#### Overrepresentation Analysis

**Figure 3:**
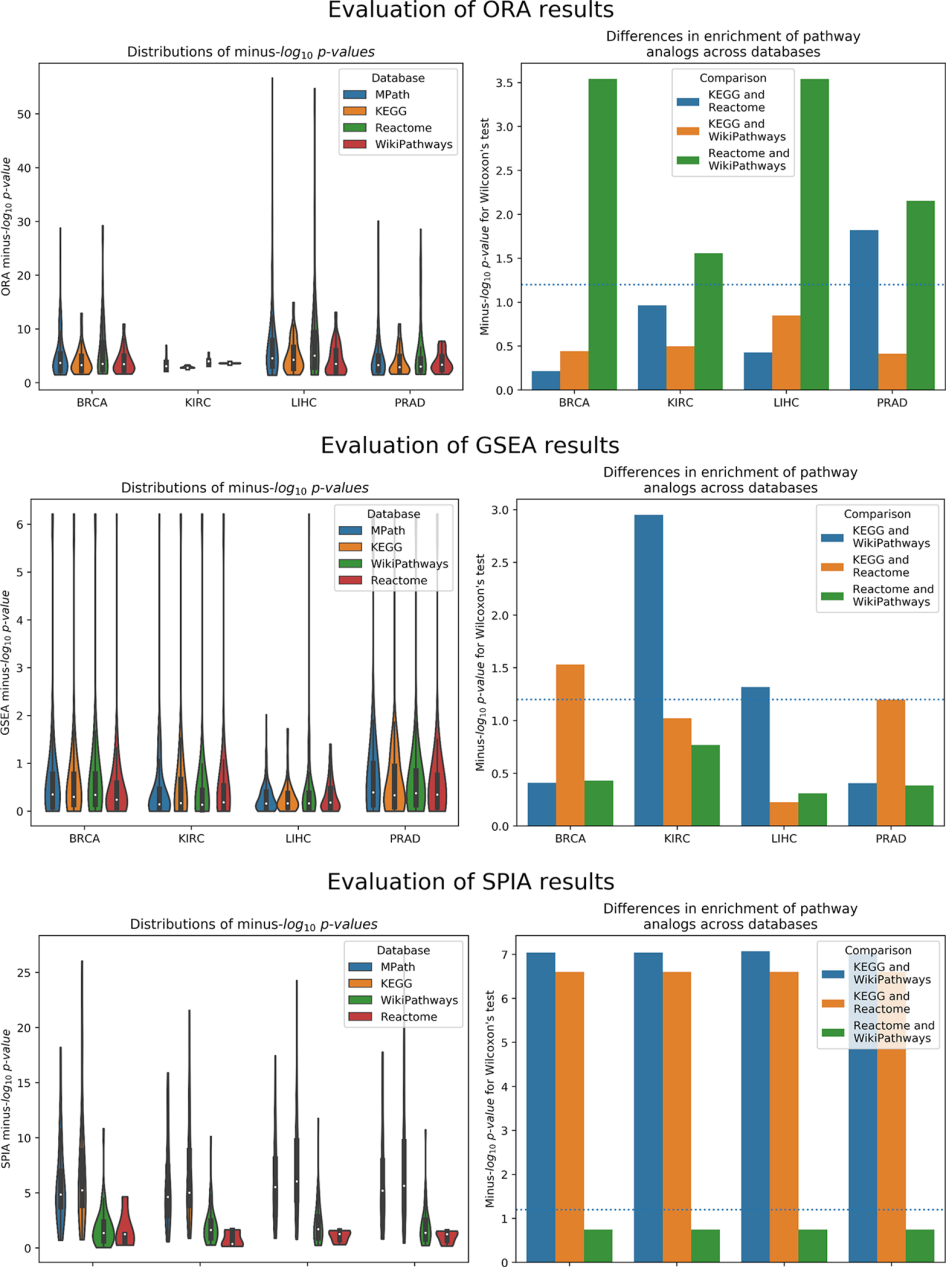
*Left* Distribution of raw *p* values of pathway analogs across databases [*top to bottom*: overrepresentation analysis (ORA), gene set enrichment analysis (GSEA), and signaling pathway impact analysis (SPIA)]. *Right* Significance of average rank differences of pathway analogs across pairwise database comparisons for the given method.

As illustrated by our results, pathway analogs from different pathway databases in several cases showed clearly significant rank differences (**Figure 3**). These differences were most pronounced between Reactome and WikiPathways. For example, while the “Thyroxine Biosynthesis” pathway was highly statistically significant (*q* value <0.01) in the LIHC dataset for Reactome, its analogs in WikiPathways (i.e., “Thyroxine (Thyroid Hormone) Production”) and KEGG (i.e., “Thyroid Hormone Synthesis”) were not. However, the pathway was found to be significantly enriched in MPath. Such differences were similarly observed for the “Notch signaling” pathway in the PRAD dataset, in which the pathway was highly statistically significant (*q* value <0.01) for Reactome and MPath, but showed no statistical significance for KEGG and WikiPathways. Similar cases were systematically observed for additional pathway analogs and super pathways, demonstrating that marked differences in rankings can arise depending on the database used.

#### Gene Set Enrichment Analysis

Similar to ORA, GSEA showed significant differences between pathway analogs across databases in several cases (**Figure 3**). These differences were most pronounced between KEGG and WikiPathways in the KIRC and LIHC datasets and between KEGG and Reactome in the BRCA and PRAD datasets. Since GSEA calculates the observed direction of regulation (e.g., over/underexpressed) of each pathway, we also examined whether super pathways or pathway analogs exhibited opposite signs in their normalized enrichment scores (NES) (e.g., one pathway is overexpressed while its equivalent pair is underexpressed). As an illustration, GSEA results of the LIHC dataset revealed the contradiction that the “DNA replication” pathway, one of 26 super pathways, was overexpressed according to Reactome and underexpressed according to KEGG and WikiPathways, though the pathway was not statistically significant for any of these databases. However, the merged “DNA replication” pathway in MPath appeared as significantly underexpressed. Similarly, in the BRCA dataset, the WikiPathways definition of the “Notch signaling” and “Hedgehog signaling” pathways were significantly overexpressed, while the KEGG and Reactome definitions were insignificantly overexpressed. Interestingly, both the merged “Notch signaling” and merged “Hedgehog signaling” pathways appeared as significantly underexpressed (*q* < 0.05) in MPath.

#### Signaling Pathway Impact Analysis

The final of the three statistical enrichment analyses conducted revealed further differences between pathway analogs across databases. As expected, differences in the results of analogous pathways were exacerbated on topology-based methods compared with ORA and GSEA, as these latter methods do not consider pathway topology (i.e., incorporation of pathway topology introduces one extra level of complexity, leading to higher variability) (**Figure 3**). Beyond a cursory inspection of the statistical results, we also investigated the concordance of the direction of change of pathway activity (i.e., activation or inhibition) for equivalent pathways. We found that for two database (i.e., LIHC and KIRC), the direction of change was inconsistently reported for the “TGF beta signaling” pathway, depending on the database used (i.e., the KEGG representation was activated and the WikiPathways one inhibited). A similar effect was observed in the “Estrogen signaling pathway,” found to be inhibited in KEGG and activated in WikiPathways in the LIHC dataset. The merging of equivalent pathway networks resulted in the observation of inhibition for both the “TGF beta signaling” and “Estrogen signaling” pathways in MPath results.

### Benchmarking the Impact on Predictive Modeling

#### Prediction of Tumor vs. Normal

**Figure 4:**
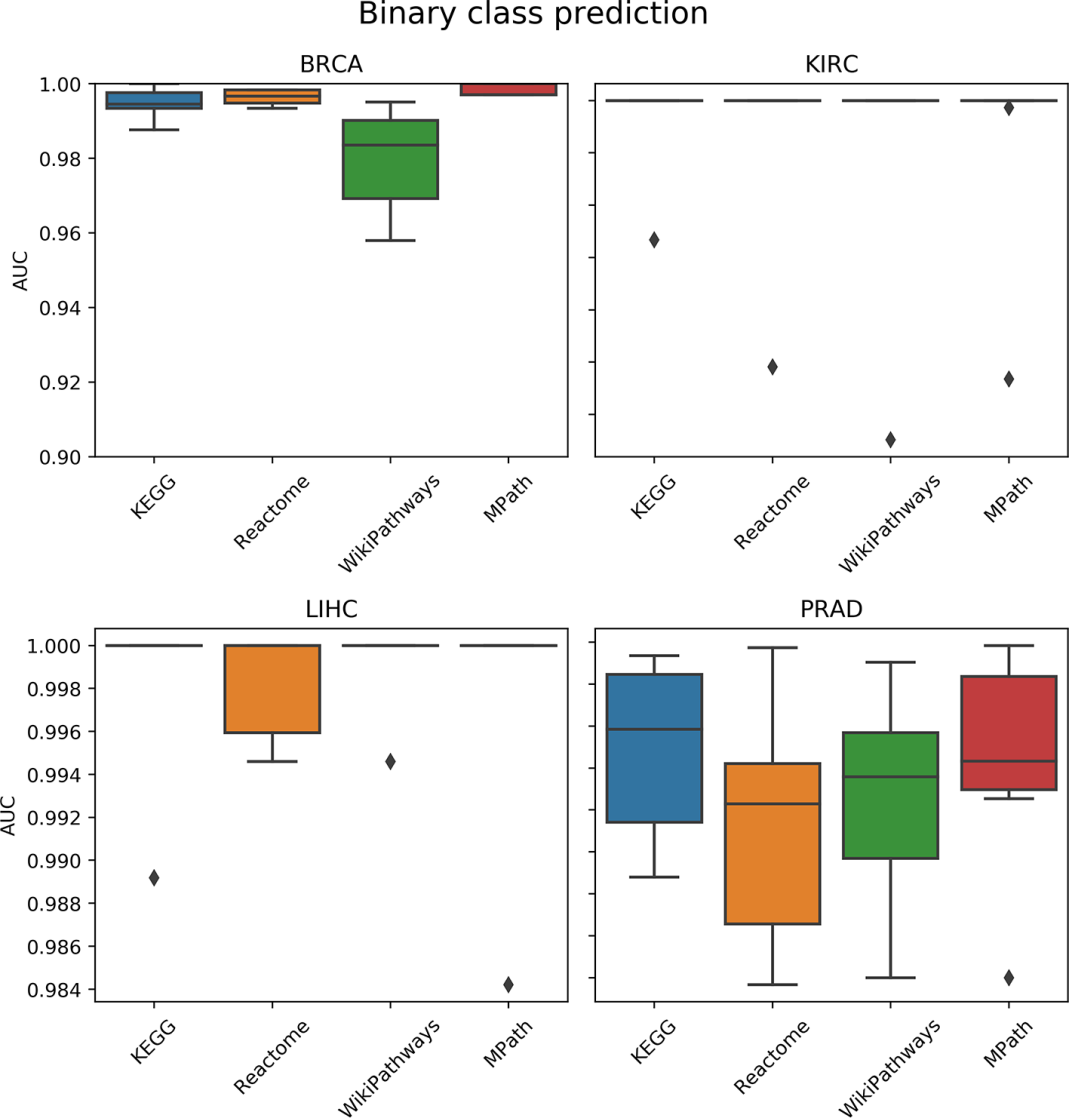
Comparison of prediction performance of an elastic net classifier (tumor vs. normal) using single-sample gene set enrichment analysis (ssGSEA)-based pathway activity profiles computed from different resources. Each box plot shows the distribution of the area under the ROC curves (AUCs) over 10 repeats of the 10-fold cross-validation procedure.

We compared the prediction performance of an elastic net penalized logistic regression classifier to discriminate normal from cancer samples based on their pathway activity profiles. The cross-validated prediction performance was measured *via* the AUC and precision-recall curve (see the corresponding *Materials and Methods* section). The AUC indicated no overall significant effect of the choice of pathway database on model prediction performance (*p* = 0.5, ANOVA *F* test; **Figure 4**). Similarly, the results of the precision-recall curve did not show a significant effect of the database selected on the model’s predictive performance. Finally, these results were not surprising due to the relative ease of the classification task (i.e., all AUC values were close to 1).

#### Prediction of Tumor Subtype

**Figure 5:**
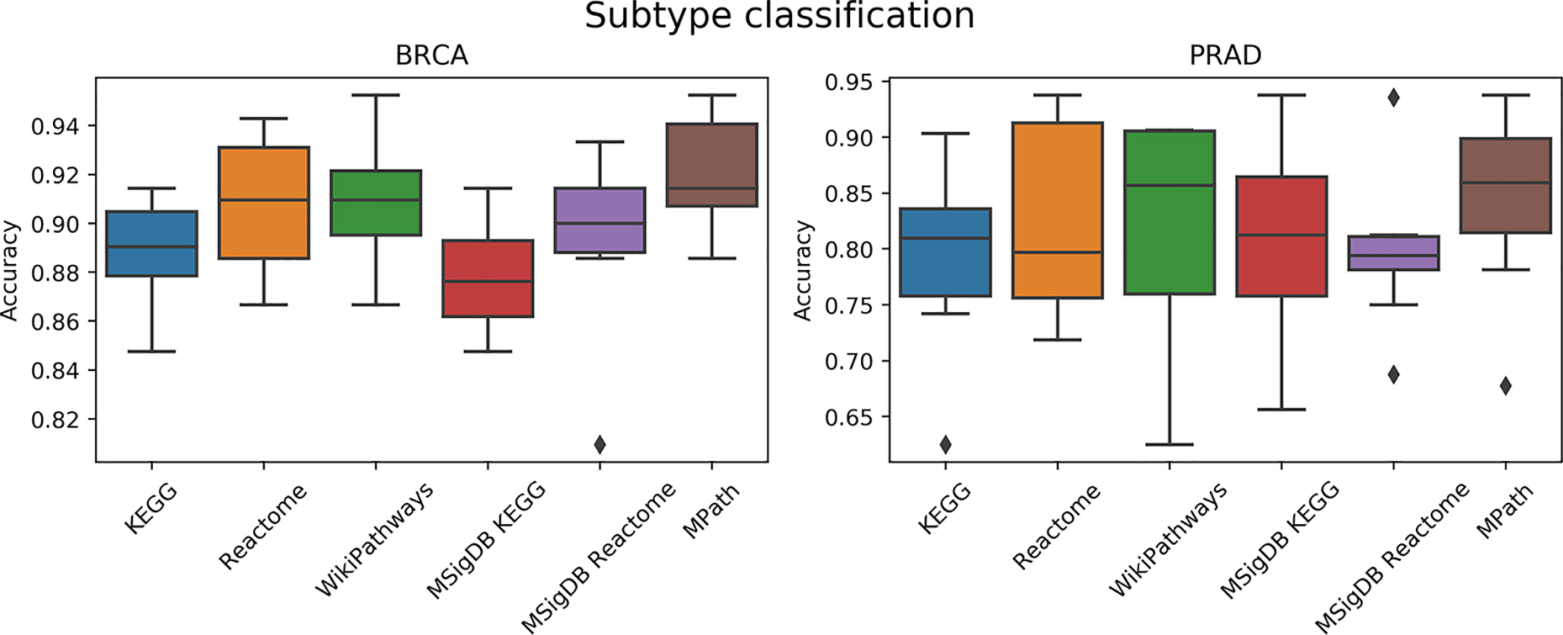
Comparison of prediction performance of an elastic net classifier (BRCA and PRAD subtypes) using single-sample gene set enrichment analysis (ssGSEA)-based pathway activity profiles computed from different resources. Each box plot shows the distribution of the area under the ROC curves (AUCs) over 10 repeats of the 10-fold cross-validation procedure.

We next compared the prediction performances of a multi-class classifier predicting known tumor subtypes of BRCA and PRAD using ssGSEA-based pathway activity profiles. **Figure 5** demonstrated no overall significant effect of the choice of pathway database (*p* = 0.16, ANOVA *F* test). We used Wilcoxon tests to investigate if each pair of distributions of the accuracies based on each database were different, but did not achieve statistical significance (*q* < 0.01) after Benjamini–Hochberg correction for multiple hypothesis testing. While the lack of significance is probably due to the limited amount of datasets (only two contained subtype information) and measurements, we would like to note that MPath showed the best classification metrics (similar to the previous classification task).

#### Prediction of Overall Survival

**Figure 6:**
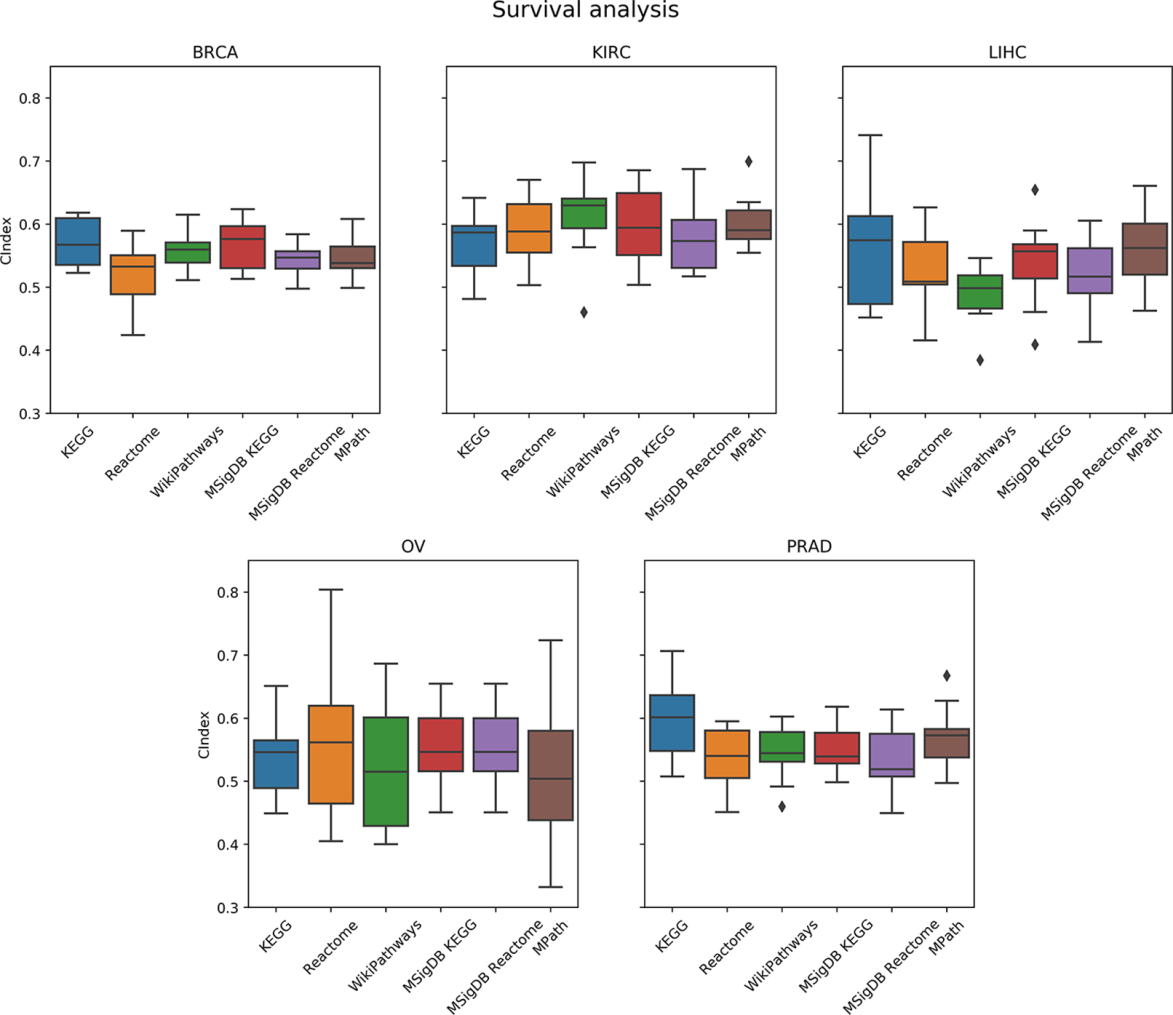
Comparison of prediction performance of an elastic net penalized Cox regression model (overall survival) using single-sample gene set enrichment analysis (ssGSEA)-based pathway activity profiles computed from different resources. Each box plot shows the distribution of the area under the ROC curves (AUCs) over 10 repeats of the 10-fold cross-validation procedure.

As a next step, we compared the prediction performance of an elastic net penalized Cox regression model for overall survival using ssGSEA-based pathway activity profiles derived from different resources. As indicated in **Figure 6**, no overall significant effect of the actually used pathway database could be observed (*p* = 0.28, ANOVA *F* test). A limiting factor of this analysis is the fact that overall survival can generally only be predicted slightly above chance level (c-indices range between 55% and 60%) based on gene expression alone, which is in agreement with the literature (Van Wieringen et al., 2009; Fröhlich, 2014; Mayr and Schmid, 2014; Zhang et al., 2018).

## Discussion

In this work, we presented a comprehensive comparative study of pathway databases based on functional enrichment and predictive modeling. We have shown that the choice of pathway database can significantly influence the results of statistical enrichment, which raises concerns about the typical lack of consideration that is given to the choice of pathway resource in many gene expression studies. This finding was specifically pronounced for SPIA because this method is a topology-based enrichment approach and therefore expected to be most sensitive to the actual definition of a pathway. At the same time, we observed that an integrative pathway resource (MPath) led to more biologically consistent results and, in some cases, improved prediction performance.

Generating a merged dataset such as MPath is non-trivial. We purposely restricted this study to three major pathway databases because of the availability of inter-database pathway mappings and pathway networks from our previous work which enabled conducting objective database comparisons. The incorporation of additional pathway databases into MPath would first require the curation of pathway mappings prior to conducting the benchmarking study, which can be labor-intensive. Furthermore, performing the tasks described in this work comes with a high computational cost (**Table 1**).

Our strategy to build MPath is one of many possible approaches to integrate pathway knowledge from multiple databases. Although alternative meta-databases such as Pathway Commons and MSigDB do exist, the novelty of this work lies in the usage of mappings and harmonized pathway representations for generating a merged dataset. While we have presented MPath as one possible integrative approach, alternative meta-databases may be used, but would require that researchers ensure that the meta-databases’ contents are continuously updated (Wadi et al., 2016).

Our developed mapping strategy between different graph representations of analogous pathways enabled us to objectively compare pathway enrichment results that otherwise would have been conducted manually and subjectively. Furthermore, they allowed us to generate super pathways inspired by previous approaches that have shown the benefit of merging similar pathway representations (Doderer et al., 2012; Vivar et al., 2013; Belinky et al., 2015; Stoney et al., 2018; Miller et al., 2019). In this case, this was made possible by the fully harmonized gene sets and networks generated by our previous work, ComPath and PathMe. A detailed description of the ComPath and PathMe publications, source code, and extensions to existing analyses (i.e., SPIA) to better suit the methods used in this work can be found in the **Supplementary Text**.

One of the limitations of this work is that we restricted the analysis to five cancer datasets from TCGA and we did not expand it to other conditions besides cancer. The use of this disease area was mainly driven by the availability of data and the corresponding possibilities to draw statistically valid conclusions. However, we acknowledge the fact that data from other disease areas may result in different findings. More specifically, we believe that a similar benchmarking study based on data from disease conditions with an unknown pathophysiology (e.g., neurological disorders) may yield even more pronounced differences between pathway resources. Additionally, further techniques for gene expression-based pathway activity scoring could be incorporated, such as Pathifier or SAS (Drier et al., 2013; Lim et al., 2016).

## Data Availability Statement

All datasets generated/analyzed for this study are included in the article/**Supplementary Material**.

## Author Contributions

DD-F conceived and designed the study. SM and DD-F conducted the main analysis and implemented the Python package. HF supervised methodological aspects of the analysis. CH and AG assisted technically in the analysis of the results. MH-A acquired the funding. SM, HF, CH, MH-A, and DD-F wrote the paper.

## Funding

This work was supported by the EU/EFPIA Innovative Medicines Initiative Joint Undertaking under AETIONOMY (grant number 115568), resources of which are composed of financial contribution from the European Union’s Seventh Framework Programme (FP7/2007-2013) and EFPIA companies in kind contribution.

## Conflict of Interest

HF received salaries from UCB Biosciences GmbH. UCB Biosciences GmbH had no influence on the content of this work.

The remaining authors declare that the research was conducted in the absence of any commercial or financial relationships that could be construed as a potential conflict of interest.
