# Corrigendum: The Impact of Pathway Database Choice on Statistical Enrichment Analysis and Predictive Modeling

**DOI:** 10.3389/fgene.2020.00436

**Published:** 2020-04-29

**Authors:** Sarah Mubeen, Charles Tapley Hoyt, André Gemünd, Martin Hofmann-Apitius, Holger Fröhlich, Daniel Domingo-Fernández

**Affiliations:** ^1^Department of Bioinformatics, Fraunhofer Institute for Algorithms and Scientific Computing (SCAI), Sankt Augustin, Germany; ^2^Bonn-Aachen International Center for IT, Rheinische Friedrich-Wilhelms-Universität Bonn, Bonn, Germany

**Keywords:** pathway enrichment, benchmarking, databases, machine learning, statistical hypothesis testing

In the original article, the correspondence email was incorrect. The correct one should be daniel.domingo.fernandez@scai.fraunhofer.de.

The authors apologize for the error and state that this does not change the scientific conclusions of the article in any way. The original article has been updated.

