# Supplementary material for: The Impact of Pathway Database Choice on Statistical Enrichment Analysis and Predictive Modeling
Source: Front Genet. 2019 Nov 22;10:1203. doi: 10.3389/fgene.2019.01203 (PMC6883970; doi:10.3389/fgene.2019.01203)
Supplement: Supplementary file 1 [file DataSheet_1.pdf]

Supplement Material

Pathway Resource	Pathways
**KEGG**	330
**Reactome**	2,219
**WikiPathways**	511
**MPath**	2,896
**MSigDB KEGG**	186
**MSigDB Reactome**	674

Table S1. Statistics of the six pathway resources used in this work. The statistics for KEGG, Reactome, WikiPathways and MPath correspond to the number of pathways retrieved from ComPath on the 28th of February, 2019. Gene sets from MSigDB correspond to the 6.2 release (July 2018).

Method	Parameters
GSEA	method=ratio_of_classes<br>number of permutations=500<br>permutation_type=phenotype<br>min_size=15/max_size=3000
ssGSEA	method=rank<br>min_size=15/max_size=3000

Table S2. Parameters used in functional class enrichment methods.

Analysis	Package	Language	Source Code
**Over-representation analysis**	statsmodels	Python	https://github.com/statsmodels/statsmodels
**GSEA**	gseapy	Python	https://github.com/zqfang/gseapy
**ssGSEA**	gseapy	Python	https://github.com/zqfang/gseapy
**Prediction tasks**	scikit-learn	Python	https://github.com/scikit-learn/scikit-learn
**Survival analysis**	scikit-survival	Python	https://github.com/sebp/scikit-survival
**SPIA**	PyBEL-Tools<br>spia	Python<br>R	https://github.com/pybel/pybel-tools<br>https://bioconductor.org/packages/release/bioc/html/SPIA.html

Table S3. Analytical packages wrapped by the `pathway_forte` package.

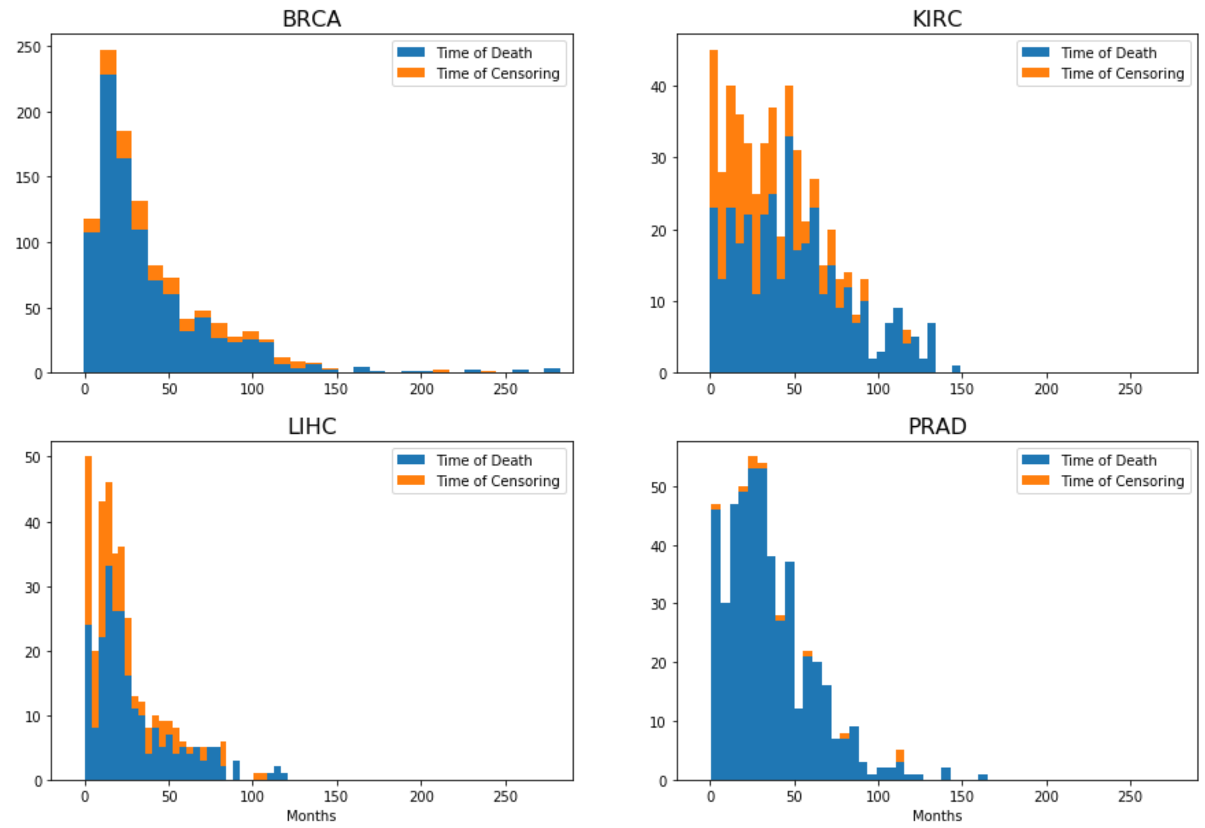

Figure S1. Histogram of the lifetime data for four TCGA datasets used for survival analysis (i.e., BRCA, LIHC, KIRC and PRAD). This visualization can be reproduced using the following Jupyter notebook:https://nbviewer.jupyter.org/github/pathwayforte/results/blob/master/notebooks/others/prediction/survival/survival_curves.ipynb.

Adapting BEL for SPIA analysis

This section outlines the transformation from the pathway BEL networks to SPIA. To conduct this transformation, we implemented a new module in the PathMe package called "spia". Therefore the SPIA files can be regenerated in the future by running a simple command: "python -m pathme export spia" (assuming that the BEL files are already parsed). Similar to the `graphite` R package, PathMe processes the networks in a way that complex nodes (i.e., reactions and complexes) are flattened and proteins and RNAs are collapsed to genes as well as variants. For further details, we defer to the PathMe paper. Specifically for Reactome, we used its hierarchy to infer the networks of the pathways containing children. The resulting BEL networks are then converted into a connectivity matrix filled with 1s in the case that there exists a relationship between two nodes, or 0s otherwise. This connectivity matrix spans over multiple relationships (thus, has multiple dimensions) that are mapped directly from BEL to the SPIA custom format (see PyBEL-Tools SPIA module for more details).

Data retrieval using ComPath and PathMe

This section describes two different approaches, ComPath and PathMe, used for pathway knowledge harmonization. The ComPath (Domingo-Fernández et al., 2018) ecosystem is a framework for integrating pathway and gene set databases. In this previously published work, we used a common approach for evaluating pathway similarity based on gene sets to support the curation of pathway mappings between each of the three

major databases (i.e., KEGG, Reactome, and WikiPathways). We focused on identifying equivalence and hierarchical relationships (e.g., Pathway A in KEGG is equivalent to Pathway B in Reactome). The second approach, PathMe (Domingo-Fernández et al., 2019), harmonizes pathway databases into BEL as a common representation schema. This approach acquires gene sets from the three major databases (i.e., KEGG, Reactome, and WikiPathways), as well as other entity classes (e.g., metabolites, miRNAs, etc.) and topological information (e.g., activation, inhibition). To combine the three pathway databases into a common format, semantics of the entities and their relationships across databases are normalized.
